# Correction of Fanconi Anemia Mutations Using Digital Genome Engineering

**DOI:** 10.3390/ijms23158416

**Published:** 2022-07-29

**Authors:** Christopher J. Sipe, Mitchell G. Kluesner, Samuel P. Bingea, Walker S. Lahr, Aneesha A. Andrew, Minjing Wang, Anthony P. DeFeo, Timothy L. Hinkel, Kanut Laoharawee, John E. Wagner, Margaret L. MacMillan, Gregory M. Vercellotti, Jakub Tolar, Mark J. Osborn, R. Scott McIvor, Beau R. Webber, Branden S. Moriarity

**Affiliations:** 1 Department of Pediatrics, University of Minnesota, Minneapolis, MN 55455, USA; sipex027@umn.edu (C.J.S.); klues009@umn.edu (M.G.K.); binge042@umn.edu (S.P.B.); wslahr@umn.edu (W.S.L.); andr0614@umn.edu (A.A.A.); wang5229@umn.edu (M.W.); apdefeo@umn.edu (A.P.D.); hinke002@umn.edu (T.L.H.); laoha002@umn.edu (K.L.); wagne002@umn.edu (J.E.W.); macmi002@umn.edu (M.L.M.); tolar003@umn.edu (J.T.); osbor026@umn.edu (M.J.O.); mcivor@umn.edu (R.S.M.); 2 Masonic Cancer Center, University of Minnesota, Minneapolis, MN 55455, USA; 3 Center for Genome Engineering, University of Minnesota, Minneapolis, MN 55455, USA; 4 Stem Cell Institute, University of Minnesota, Minneapolis, MN 55455, USA; 5 Medical Scientist Training Program, University of Washington, Seattle, WA 98195, USA; 6 Division of Blood and Marrow Transplantation, University of Minnesota, Minneapolis, MN 55455, USA; 7 Division of Hematology, Oncology and Transplantation, University of Minnesota, Minneapolis, MN 55455, USA; verce001@umn.edu

**Keywords:** Fanconi anemia (FA), gene therapy, base editing, adenine base editing (ABE), cytosine base editing (CBE), CRISPR-Cas9, digital genome engineering, double strand breaks, bone marrow failure, base excision repair, Fanconi anemia repair pathway

## Abstract

Fanconi anemia (FA) is a rare genetic disease in which genes essential for DNA repair are mutated. Both the interstrand crosslink (ICL) and double-strand break (DSB) repair pathways are disrupted in FA, leading to patient bone marrow failure (BMF) and cancer predisposition. The only curative therapy for the hematological manifestations of FA is an allogeneic hematopoietic cell transplant (HCT); however, many (>70%) patients lack a suitable human leukocyte antigen (HLA)-matched donor, often resulting in increased rates of graft-versus-host disease (GvHD) and, potentially, the exacerbation of cancer risk. Successful engraftment of gene-corrected autologous hematopoietic stem cells (HSC) circumvents the need for an allogeneic HCT and has been achieved in other genetic diseases using targeted nucleases to induce site specific DSBs and the correction of mutated genes through homology-directed repair (HDR). However, this process is extremely inefficient in FA cells, as they are inherently deficient in DNA repair. Here, we demonstrate the correction of *FANCA* mutations in primary patient cells using ‘digital’ genome editing with the cytosine and adenine base editors (BEs). These Cas9-based tools allow for C:G > T:A or A:T > C:G base transitions without the induction of a toxic DSB or the need for a DNA donor molecule. These genetic corrections or conservative codon substitution strategies lead to phenotypic rescue as illustrated by a resistance to the alkylating crosslinking agent Mitomycin C (MMC). Further, FANCA protein expression was restored, and an intact FA pathway was demonstrated by downstream FANCD2 monoubiquitination induction. This BE digital correction strategy will enable the use of gene-corrected FA patient hematopoietic stem and progenitor cells (HSPCs) for autologous HCT, obviating the risks associated with allogeneic HCT and DSB induction during autologous HSC gene therapy.

## 1. Introduction

Fanconi anemia (FA) is a rare genetic disease affecting 1:100,000 live births in the US annually. FA is characterized by bone marrow failure (BMF) and a predisposition to malignancies, such as acute myeloid leukemia (AML) [1]. Solid tumors are also common, predominantly head and neck squamous cell carcinoma (HNSCC) and gynecologic cancers (vulvar and cervical) [2]. Additional manifestations include pancytopenia, developmental delay, osteoporosis, and congenital malformations [3]. These complications arise from a defective FA pathway which participates in multiple DNA damage repair and surveillance mechanisms, such as the interstrand crosslink (ICL) repair pathway, homologous recombination (HR), non-homologous end joining (NHEJ), translesion synthesis (TLS), and replication fork protection [4]. The FA pathway/complex is comprised of 23 known genes, including *FANCA*, *FANCC*, *FANCD1* (BRCA2), *FANCD2*, *FANCG*, *FANCN* (PALB2), *FANCO* (RAD51C), *FANCQ* (ERCC4/XPF), *FANCR* (RAD51), *FANCS* (BRCA1), and *FANCV* (REV7) [5,6,7]. Mutations in any of these genes can lead to different disease states with a wide range of clinical presentations; however, ~70% of FA cases are caused by mutations on *FANCA* [8]. The accumulation of unrepaired ICLs from endogenous and exogenous sources such as aldehydes, reactive oxygen species (ROS), or alkylating agents such as Mitomycin C (MMC), are the driving forces of disease progression and phenotypic presentation [5]. These unchecked genetic stressors often lead to p53- and p21-induced cell cycle arrest, particularly in the bone marrow compartment [6].

Treatment by allogeneic hematopoietic stem cell transplant (HCT) can be curative for the lethal BMF and hematological malignancy manifestations of the disease (i.e., severe aplastic anemia, myelodysplasia, leukemia); however, allogeneic HCT in FA patients is associated with significant risks of debilitating morbidities and mortality due to side effects from chemo(radio)therapy conditioning and the immunological complications of allogeneic HCT, such as graft-versus-host disease (GvHD) and opportunistic infections from prolonged immune incompetence [7]. In addition, there appears to be a magnification of cancer risk [9] after allogeneic HCT relative to patients without a history of HCT. The timing of transplant is critical, necessitating close monitoring in order to maximize success, e.g., prior to severe marrow failure and risks of systemic infection, and leukemia transformation [10]. Preconditioning regimens are generally aimed at preparing the bone marrow niche for transplant; however, increased sensitivity of FA cells to alkylating agents and total body irradiation (TBI), potentially leading to widespread DNA damage beyond the hematopoietic compartment, have led to regimens tailored to FA [10,11].

While autologous HCT with gene-corrected HSCs bypasses many of these issues, as it may reduce the need for toxic conditioning and eliminate the immunological risks of allogeneic HCT, low numbers of HSPCs are available for genetic modification even when blood counts are relatively normal. In addition, there are risks of cellular toxicities and insertional mutagenesis with lentiviral complementation approaches, and some form of conditioning may be required to enhance the competitive repopulation relative to host FA HSCs. High costs associated with developing GMP-compatible viral vectors for each of the 23 FA genes for lentiviral transfer also remains a significant hurdle.

Several recent preclinical studies have aimed to insert functional copies of FA genes (i.e., complementation) through homology-directed repair (HDR) in FA patient primary cells. Unfortunately, these studies produced low initial insertion/correction as FA cells have difficulty resolving the DSBs that nucleases induce (Figure 1A). The targeting of FANCC in patient-derived fibroblasts with CRISPR-nCas9 and an exon 4 cDNA plasmid construct produced ~2% gene-corrected cells [12], while the targeting of FANCD1 in patient-derived fibroblasts using CRISPR-Cas9 with a dsDNA template showed that 4.5% of cells underwent HDR [13]. A different strategy aimed to create a frameshift restoration in the *FANCA* c.295 C >T FA patient lymphoblastoid cell line (LCL) using CRISPR-Cas9-induced NHEJ created a 21 base pair deletion, allowing the production of the functional FANCA protein as shown by FANCD2 foci formation and resistance to MMC; however the initial therapeutic insertions/deletions (indels) were as low as 2.3% [14].

In the last 20 years there have been four clinical trials of FA gene therapy [15]. The first three of these studies have included nine total patients that were successfully transplanted, all using lentiviral *FANCA* complementation constructs in patients with varying *FANCA* mutations. The most recent of the three trials tested viral complementation of *FANCA* in autologous HSPCs using a lentiviral cDNA vector (NCT02931071) [16]. Four patients with mild to moderate BMF were treated, with a minimum follow-up of three years after gene therapy. The phenotypic correction of blood and bone marrow cells was demonstrated by the acquired resistance of HSPCs to clastogenic agents. None of the patients have yet demonstrated improved blood counts, although some patients have not had further disease progression [17]. Additionally, the peripheral blood (PB) cell counts remained in the moderate BMF range, raising concerns about long-term efficacy. The second trial involved two patients infused with HSPCs transduced using a lentiviral vector encoding a *FANCA* expressing cDNA (MSCV-FANCA) (NCT00272857) [18]. At six months post-transplant, blood counts had increased moderately with no adverse events in either patient, however longer-term follow-up is necessary to gauge efficacy [15]. The third trial (NCT01221018) included two patients, both of whom tolerated treatment well with no adverse events reported, but low and declining levels of transduced cells were observed in the peripheral blood [19,20]. The final ongoing trial from Rocket Pharmaceuticals involves nine patients (and three additional patients with less than 12 months follow-up) who underwent autologous engraftment of HSPCs that were transduced with a lentiviral vector carrying *FANCA*. The survival of BM colony-forming units (CFU) at 12–36 months post infusion has reached an average of 49% survival after treatment with ICL agents (NCT04248439) [19].

The overall success of these trials remains unsatisfactory and highlights the need for alternative approaches. Low transduction efficiency, the paucity of corrected cells in the infusion product, and the absence of conditioning regimens all likely play a role in these observations. Thus, while clinical trials are in place that utilize gene therapy approaches targeting FA patient HPSCs [15], we hypothesized that the least invasive and most effective way to accomplish genetic correction in FA HSPCs is to deploy digital editing technologies that carry out gene editing without DSBs, the need for DNA donor molecules, or the need to engage homology-directed DNA repair machinery.

Cas9 base editors (BEs) utilize a Cas9 nickase (nCas9) fused to a deaminase that enzymatically converts cytidine to uridine (cytidine base editor, CBE) or adenosine to inosine (adenosine base editor, ABE), leading to enzymatic and site-specific C > T or A > G base transition upon DNA replication or broad cellular repair, respectively (Figure 1B,C) [21,22]. Specific to CBE, a uracil glycosylase inhibitor (UGI) is also fused to the nCas9 which blocks DNA surveillance mechanisms from excising and repairing this deamination back to the wild-type sequence [23]. Previously, we optimized the use of BEs in primary human T cells, achieving high levels of multiplex editing with low to no detectable off-target editing, no translocations between DSB sites, and no impact on T cell expansion or cytotoxic function [20]. Moreover, there is no need for a DNA donor molecule, and critical for this application of correcting FA HSPCs, BEs do not require DSBs, as both DNA and DSBs are known to be highly toxic to FA primary stem cells and can drive genetic instability and induce cell cycle arrest and cancer [24]. Furthermore, the induction of p53 [25], complex chromosomal rearrangements, chromothripsis [26], and large DNA deletions [27] have all been observed following the use of nucleases to carry out HDR. These issues are also of greater concern in the setting of transplanted HSPCs that give rise to many cells across multiple lineages for the lifetime of the recipient. For these reasons, we deployed digital genome editing technologies in FA patient primary cells for the first time to achieve precise and highly efficient mutation correction.

Here, we corrected or restored the reading frame of two independent *FANCA* patient mutations using CBE and ABE, respectively, which conferred a phenotypic rescue as evidenced by desensitization to MMC, indicating the restoration of the cell’s ability to overcome ICLs and subsequent DSBs. CBE was used to restore a wild-type (WT) splice donor (SD) site in primary FA patient cells, and ABE was used to eliminate a premature STOP codon (pmSTOP) by restoring the reading frame in primary FA patient cells containing the Spanish Romani Founder mutation (1:64 carrier frequency), which is the most common FA mutation worldwide [28]. Base editing resulted in the restoration of FANCA protein expression, and a functional FA pathway as evidenced by downstream FANCD2 monoubiquitination after challenge with ICL alkylating agents. This base editing strategy will enable the use of gene-corrected FA patient HSPCs for autologous HCT and thereby eliminate the risks of allogeneic HCT.

## 2. Results

### 2.1. Base Editors Are Highly Functional in FA Cells

As previous work established that FA cells have impaired DNA repair and surveillance mechanisms in addition to base excision repair (BER) and mismatch repair (MMR) [29], we first set out to determine if BE-induced deamination is functional in FA cells. The major histocompatibility complex I (MHCI) structural component beta-2 microglobulin (*B2M*) is expressed on the surface of nucleated cells and serves as an excellent positive control for base editing. Using CBE and ABE, we targeted the splice donor (SD) site immediately downstream of *B2M* exon 1 (position C6 of the editing window), which we have shown results in high levels of base editing and subsequent protein knockout (KO) (Figure 2A) [20]. Electroporating healthy donor (HD) T cells with *B2M* Ex.1 SD sgRNA and CBE or ABE mRNA resulted in editing rates of 95% and 92%, respectively (Figure 2B). In FA patient primary fibroblasts, high editing rates at *B2M* were also achieved, albeit at slightly lower levels (85% with CBE and 60% with ABE) (Figure 2C). These results confirm that BEs do not require a fully intact FA pathway to operate and that they can perform at high levels, as demonstrated in previous reports at the *B2M* locus [30].

### 2.2. Genetic Correction with Base Editor Confers Phenotypic Rescue of FA Cells

FA patient samples containing a *FANCA* c.3934 + 2T > C mutation have a single T > C substitution at the +2 position of an SD site on exon 39 of *FANCA* leading to loss of protein expression (Figure 3A). We hypothesized this mutation could be directly corrected back to WT by restoring the SD sequence using CBE. FA patient LCL were first generated from the patient’s peripheral blood mononuclear cells (PBMCs) (Supplementary Figure S1A,B), and electroporated with *B2M* Ex.1 SD sgRNA in combination with CBE, ABE or Cas9 mRNA to reconfirm the functionality of base editing in a separate FA patient line. This strategy produced editing levels of 61% and 65% for CBE and ABE, respectively, and indel rates of 38% with Cas9 nuclease (Figure 3B,C). Each enzyme produced similar editing or indel rates in FA patient LCL as compared to FA patient fibroblasts (data not shown). B2M protein was subsequently assessed by flow cytometry to confirm KO, further demonstrating the effectiveness of base editing in cells with varying FA mutations. Cell surface B2M protein KO reached 40% for CBE, 70% for ABE, and 50% for Cas9 (Figure 3D, Supplemental Figure S2A–D).

Next, FA patient fibroblasts were electroporated with BE4 mRNA in combination with a sgRNA containing a 20 nucleotide (nt) sequence targeting the *FANCA* c.3934 + 2T > C mutation. Initial editing efficiencies of the target (C4) base were low (<5%), but detectable, indicating this strategy is feasible but requires optimization. Previous reports have suggested that extending the sgRNA length can shift the editing window to place the desired nucleotide in a more optimal position for higher base editing [31]. Three additional sgRNAs were thus designed ranging from 21 to 23 nt in length by adding bases on the 5′ end, which shifted the target C base from position 4 to positions 5, 6, and 7. Notably, previous reports indicate that positions 5, 6, and 7 are subject to the highest rates of deamination with CBE [22,32].

To test extended nucleotide sgRNAs ranging in length from 20 to 23 nt, we conducted an in vitro base editing assay. PCR amplicons of the *FANCA* c.3934 locus were incubated with ribonucleoprotein (RNP) comprising BE4 protein and each of the varying nucleotide length sgRNAs, respectively. Consistent with initial fibroblast editing, Sanger sequencing of the PCR amplicons revealed low level C > T editing (<5%). Extending the sgRNA to 22 and 23 nucleotides shifted the editing window and increased C > T editing to >20%, consistent with observations from previous reports (Figure 3E) [31]. This observation represents an optimization strategy that can be used to shift editing windows based on protospacer adjacent motif (PAM) availability to increase editing rates. Unexpectedly, electroporating fibroblasts with this *FANCA* c.3934 + 2T > C 22 nt sgRNA and CBE mRNA again produced editing levels < 5%. However, after ~50 days in culture, the corrected fibroblasts expanded and reached edited levels of 26% demonstrating the selective outgrowth of base-edited cells (Figure 3F,G). This observation represents an optimization strategy that can be used to shift editing windows based on protospacer adjacent motif (PAM) availability to increase editing rates.

To demonstrate phenotypic rescue of *FANCA* base-edited cells, fibroblasts corrected with the *FANCA* c.3934 + 2T > C 22 nt sgRNA and allowed to outgrow, along with uncorrected fibroblasts, were cultured at varying concentrations of MMC (0–80 nM). FA patient samples are highly sensitive to this alkylating agent, which induces DNA ICLs that cannot be repaired in FA cells [33]. At lethal concentrations of MMC (40 nM) [34], corrected cells showed a significant improvement in cell survival compared to uncorrected cells after 6 days (Figure 3H). These data confirm the feasibility of using BEs for correcting FA patient mutations by showing that editing is possible at the target site and that the FA pathway can then be phenotypically rescued.

### 2.3. The Most Common FA Mutation Is Highly Amenable to Base Editing

Fibroblasts are not an ideal cell source for optimizing base editing approaches given the difficulties with cell expansion and culture manipulation along with patient scarring after skin punching [36]. Thus, we continued with LCL as a superior cell source that facilitates manipulation. *FANCA* mutations more abundant in the general population were also examined to broaden the scope of base editing enzyme application to more FA patients. The most common founder mutation worldwide, found in the Spanish Romani population (1:64 carrier frequency), is ideally suited for this purpose. Therefore, we obtained patient LCLs [28] (FA-55) containing a *FANCA* c.295C > T mutation that have a single C > T substitution in exon 4 of *FANCA* causing a pmSTOP codon leading to truncated non-functional protein (Figure 4A). Unfortunately, the *FANCA* c.295C > T pmSTOP codon (TAG) cannot be directly converted back to the WT glutamine codon (CAG) using CBE or ABE technology. However, a previous study using CRISPR-Cas9 in combination with a sgRNA targeting exon 4 to induce indels led to functional protein production by removing 7 amino acids, including the pmSTOP [14]. Thus, we deployed ABE to target the adenine base of the pmSTOP codon (TAG) for conversion to guanine (TGG), resulting in a conservative substitution to tryptophan (Figure 4A). Notably, in alignment of 248 metazoan *FANCA* sequences from Ensembl, there is a diversity of amino acids present at the position in question (p. 99), specifically in the Hoffman’s Two-Toed Sloth, suggesting that a tryptophan at this position is compatible in mammals (Figure 4B).

Initial base editing frequencies after electroporation using ABE7.10max mRNA and *B2M* Ex.1 SD sgRNA in FA-55 LCL produced *B2M* editing efficiencies > 70% (Figure 4C,D). During these experiments, a highly active ABE variant (ABE8e) was reported that demonstrates dramatically enhanced rates of base editing [37]. Thus, we deployed ABE8e mRNA analogous to ABE7.10max mRNA and obtained *B2M* editing frequencies > 99% (Figure 4C,D). Targeting *FANCA* c.295C > T with sgRNA combined with ABE7.10max mRNA produced base editing rates of <10%, but dramatically increased to >70% when using ABE8e mRNA (Figure 4E,F).

To test phenotypic recovery of MMC resistance, corrected cells were cultured in 40 nM MMC or DMSO for 14 days. Due to low initial editing with ABE7.10max, cell viability decreased at day six where ICLs were initially detrimental, but recovered to levels similar to DMSO controls, indicating the outgrowth of MMC-resistant, corrected cells (Figure 5A). Further, the density and expansion of corrected cells in MMC began to rebound and approach DMSO levels and performed much better than uncorrected cells; however, the corrected cells did not perform as well as the cells treated without MMC (DMSO) (Figure 5B,C). These ABE7.10max-corrected cells successfully outgrew uncorrected cells and approached editing levels of 60% within 14 days in MMC culture (Figure 4F). Conversely, MMC exposure of the LCL corrected with ABE8e showed no effect on viability and was comparable to DMSO controls showing high level editing (>70%) equating to an immediate recovery of the FA pathway and a resistance to MMC (Figure 5A–C). The ABE8e-corrected LCL did not expand further after MMC selection, suggesting that all FA cells now contained at least one base-edited allele [38].

A western blot was then performed to show that the restoration of FANCA protein product was indeed the cause of the FA pathway reactivation and renewed resistance to MMC after BE. After LCL editing, a FANCA band was present albeit to a slightly lower intensity compared to HD controls (Figure 5D). The FA core complex containing FANCA, once activated, interacts with the FANCD2/I complex, a helicase that directly contacts the ICL DNA, aiding in creating a DSB. This is accomplished by the monoubiquitination of both FANCD2–FANCI and thus FANCD2 monoubiquitination is an indicator of an intact FA pathway [39]. Under the conditions of DMSO and MMC, a second band of slightly higher molecular weight (MW) than FANCD2 was present after LCL base editing, as with the HD controls (Figure 5E). This result indicates the monoubiquitination of FANCD2, the presence of ubiquitin (8.6 kDa), and thus an intact FA pathway. Slightly more intense bands under conditions of MMC illustrate a higher response to ICLs compared to DMSO controls.

The high efficiency of ABE8e editing taken together with a new cellular resistance to MMC, FANCA protein product restoration, and an intact FA pathway as shown by FANCD2 monoubiquitination, provides strong evidence supporting the feasibility and justification of using BEs for treating FA mutations in patient primary cells. The optimization of these reagents in patient-derived cells is an essential practice before editing patient HSPCs in the development of gene therapy using autologous corrected cells, given the paucity of FA patient HSPCs [6].

### 2.4. Prediction of FA Mutation Outcomes with Base Editors

To assess how widely digital editing could be applied to correct mutations in FA DNA repair pathway genes [5] (Supplementary Figure S3A), we analyzed all pathogenic mutations as well as those of uncertain significance documented on NCBI’s ClinVar database. Consistent with the literature, the most frequently mutated gene was *FANCA* (Supplementary Figure S3B) [40]; however, other genes were unexpectedly prominent, such as FANCP and FANCJ, which may be due to somatic mutations in cancer samples that are ambiguously labeled on ClinVar submissions (Supplementary Figure S3C) [41]. Among the ClinVar mutations, transition (41.1%) and transversion (28.8%) mutations comprised most mutations (Figure 6A). Using an algorithm to categorize these mutations (Supplementary Figure S2A), we predict that most FA mutations are correctable by CRISPR-Cas9 based genome editing without the need for DSBs. This encapsulates 45.7% of mutations possibly correctable by base editing and 43.1% possibly correctable by prime editing (PE), a technology that also utilizes a nCas9 which is fused to a reverse transcriptase (RT) allowing for correction of all 12-point mutations and large insertions and deletions (Figure 6B). Given the additional mutations that were found to be correctable with PE, future studies should also focus on the use of PE for FA gene therapy as DSBs are avoided. This leaves only 11.1% of FA mutations requiring an alternative editing strategy (Figure 6C). Further, we explored how computational prediction of base editing with the program BE-Hive [42] can be used to inform reagent selection for gene therapy. Using the *FANCA* c.3934 + 2T > C mutation as an example, we found that the target to bystander editing ratio can be improved by using alternative BEs, such as evoAPOBEC-BE4 (Figure 6D–F). Collectively, these findings show how computational methods can be used to guide candidate mutation selection and correction to increase the odds of therapeutic success when deploying digital editing (Figure 6).

## 3. Discussion

Here, we have demonstrated for the first time that FA patient primary cells are highly amenable to digital editing using BEs in a therapeutic setting. This was accomplished in an aberrant FA pathway environment, showing that this pathway does not intersect mechanistically with processes involved in base editing. This was shown in *FANCA* deficient cell lines; other genes in the FA pathway such as *FANCC* or *FANCG* may not be amenable to base editing. Further, after the mutation correction of *FANCA* c.3934 + 2T > C to c.3934 + 2C > T which led to a gained resistance to MMC, or the conservative substitution of a pmSTOP codon with *FANCA* c.295C > T to c.296A > G, the FA pathway was restored as shown by a resistance to MMC, FANCA protein restoration, and FANCD2 monoubiquitination. Previous clinical trials have shown that FA CD34+ HSPCs can be isolated, genetically complemented via viral transduction, and used for autologous HCT. The optimization of editing reagents and conditions in FA patient primary cells is essential before moving to precious patient CD34+ HSPCs, especially for FA patients as there are so few stem cells available for correction [6]. A different approach that bypasses the need for obtaining FA patient HSPCs for autologous gene therapy is to create iPSCs from primary blood cells, correct these using BE technology, and perform differentiation into an HSPC-like cell that can be engrafted into the patient for the repopulation of the entire hematopoietic system [43]. This area of research is ever-growing with goals to treat hematologic genetic diseases, as well as cancers arising from the hematopoietic system [44]. Although protocols have been described that can differentiate iPSCs into an “HSPC-like” cell which expresses many of the markers of true HSPCs, a perfect recapitulation has not been accomplished [43]. More primitive HPSCs have been documented as opposed to fully engraftable, definitive HPSCs.

Another exciting alternative to the ex vivo correction of HSPCs is in vivo delivery of gene editing reagents. In fact, there has been an explosion of research focused on the in vivo delivery of gene therapy reagents to treat many genetic diseases and cancers. Most of these studies involve the use of recombinant adeno-associated vectors (rAAVs) [45], the HDAd5/35++ vector [46], or lipid-like nanoparticles (LLNPs) [47] to package and deliver CRISPR-Cas9 and related reagents to target cells. These convenient (one-time intravenous injections) and direct in vivo approaches are ideal for genetic disorders and cancers arising from the HSPC compartment such as FA. They eliminate the need for ex vivo gene therapy manipulation, conditioning regimens, HSPC extraction, expansion, and infusion along with all associated complications. Several groups have begun to use these technologies for the delivery of BE reagents, including rAAV [48], HDAd5/35++ vector [49], LLNPs [50], and engineered virus-like particles (eVLPs) [51]. Combining in vivo gene therapy delivery to HSPCs of FA patients in combination with BE technology may represent the future of FA-related therapies and treatment. There are, however, complications associated with the in vivo delivery of genome editing reagents. To deliver reagents to cells of interest there must be some specificity of the vehicle, which requires an understanding of the biology of the target cell as well as the editing reagent capsule. In the case of rAAV vectors that have serotypes for a specific cell or cell category type, the body can have an inherent immune response to the foreign rAAV vector [52]. The delivery of BE reagents to HSPCs would potentially require the mobilization of these cells into the bloodstream from the bone marrow [53], targeting them with editing reagents and then allowing them to re-engraft into the bone marrow niche. Additionally, there are some viruses that are capable of directly honing to the bone marrow for the delivery of base editing reagents.

After successfully base editing FA patient primary cells at high rates, we analyzed all mutations in FA genes of uncertain, known pathological consequence and found that 45.7% of unique mutations can be potentially corrected by CBE or ABE. Further, an additional 43.1% of mutations are transversions, which could possibly be conservatively substituted by base editing or corrected with prime editing. Off-target effects arising at genomic regions which are highly similar to the on-target sgRNA sequence were not investigated here and represent a limitation of this study. However, given that base editing technology produces fewer guide-dependent off-target effects than CRISPR-Cas9 nuclease [54], this work still sets the stage for an important pipeline of treating different FA mutations with base editing in a safer personalized medicine approach. Future work should include these off-target analyses both in a biased approach (computationally predicted) and in an unbiased approach such as with GUIDE-Seq [55]. Further, base editing is ideally suited for correcting FA-mutated cells which are inherently deficient in DNA repair as BEs do not create DSBs. This is further illustrated by the low levels of CRISPR-Cas9 correction or gene insertion in FA mutant cells in previous work [12] and a dearth of clinical trials and data that use these DSB strategies [15]. This proposed strategy is optimal because editing reagents can be tested and optimized ex vivo from FA patient blood samples which are easy to obtain, isolate, and manipulate. After confirming that the editing strategy indicated by the prediction software does indeed translate into human cell editing, precious HSPCs can then be obtained, base edited, and reinfused back into the patient for engraftment. This personalized medicine approach tailored to each FA patient’s specific mutation does, however, have cost disadvantages. Designing, testing, optimizing, and the application of downstream approaches for the wide variety of FA mutations listed on ClinVar would likely be very costly. This is further compounded when considering that some FA patients are the only reported individual with a specific mutation in each specific FA gene [56].

## 4. Materials and Methods

### 4.1. Fibroblast Culture

Patient-derived *FANCA* c.3934 + 2T > C (rs771775516) primary fibroblasts provided by Dr. Mark J. Osborn (Department of Pediatrics, Pediatric Bone and Marrow Transplant Center, University of Minnesota, Minneapolis, MN, USA) were collected via skin biopsy. Cells were maintained in Alpha Minimum Essential Medium Eagle + GlutaMAX (αMEM, Gibco, Waltham, MA, USA, #32561-037) with HyClone Fetal Bovine Serum (20%, hFBS, R&D Systems, Minneapolis, MN, USA, #S11550), non-essential amino acids (10×, NEAA, Gibco, #11140050), antioxidant supplement (1×, Sigma-Aldrich, St. Louis, MO, USA, #A1345-5ML), Penicillin–Streptomycin (1%, 10,000 U/mL, P/S, Invitrogen, Waltham, MA, USA, #TMS-AB2-C), Epidermal Growth Factor from mouse submaxillary glands (10 ng/mL, mEGF, Sigma-Aldrich, St. Louis, MO, USA, #E4127), and basic human recombinant Fibroblast Growth Factor (0.5 ng/mL, hFGF, Sigma-Aldrich, St. Louis, MO, USA, #F0291) under hypoxic conditions (5% O_2_, 5% CO_2_, 37 °C).

### 4.2. Preparation of LCL Feeder Cells

Vials of MRC-5 feeder cells (ATCC, Manassas, VA, USA, #55-X) were resuspended in 27 mL of Iscove’s Modified Dulbecco’s Medium (IMDM, Gibco, #12440053) with HyClone Fetal Bovine Serum (20%, hFBS, R&D Systems, #S11550) and Penicillin–Streptomycin (1%, 10,000 U/mL, P/S, Invitrogen, #TMS-AB2-C) under normoxic conditions (21% O_2_, 5% CO_2_, 37 °C). Per T-25 flask, 3 mL of feeder cell suspension was plated for transfection of bulk *FANCA* c.3934 + 2T > C (rs771775516) peripheral blood mononuclear cells (PBMCs) 1–5 days post feeder cell thawing.

### 4.3. LCL Transformation

After aspirating media from feeder cells, 1 mL of Epstein–Barr virus (EBV, ATCC, #VR-1492) was added per flask with 1–5 × 10^6^ *FANCA* c.3934 + 2T > C (rs771775516) PBMCs and 3 mL of LCL media (below) under normoxic conditions (21% O_2_, 5% CO_2_, 37 °C). At 10–14 days post transformation, cells were removed from feeder cells by transferring to a new T-25 flask. Once transformation reached later stages (increased turbidity or yellowing of culture media) cells were transferred from a T-25 to T-75 flasks. Cells were kept at a density of 1–3 × 10^5^ cells/mL and split to additional T-75 flasks as needed. Once 2e7 cells were reached, aliquots were frozen at a density of 5e5 cells/mL in CryoStor CS10 (StemCell Technologies, Vancouver, CA, USA, #07930). An aliquot of 1 × 10^5^ *FANCA* c.3934 + 2T > C (rs771775516) LCLs were then stained with PE anti-human CD19 antibody clone HIB19 (BioLegend, San Diego, CA, USA, 302254) for flow cytometry.

### 4.4. LCL Culture

FA-55 patient-derived *FANCA* c.295C > T (rs1057516430) LCL provided by Dr. Paula Río (Division of Hematopoietic Innovative Therapies, Centro de Investigaciones Energéticas Medioambientales y Tecnológicas (CIEMAT), Madrid, Spain) were generated by transduction of isolated primary peripheral B cells with EBV. “FA patients were encoded to protect their confidentiality; informed consents were obtained in all cases prior to the generation and use of the cell lines” [16]. Cells were maintained in Roswell Park Memorial Institute Medium (RPMI, Gibco, Waltham, MA, USA, #11875-093), with HyClone Fetal Bovine Serum (20%, hFBS, R&D Systems, Minneapolis, MN, USA, #S11550), non-essential amino acids (10×, NEAA, Gibco, Waltham, MA, USA, #11140050), Sodium Pyruvate (1 mM, Gibco, Waltham, MA, USA, #11360-070), Penicillin–Streptomycin (1%, 10,000 U/mL, P/S, Invitrogen, Waltham, MA, USA, #TMS-AB2-C), and 2-Mercaptoethanol (0.005 mM, BME, Gibco, Waltham, USA, #21985-023) under normoxic conditions (21% O_2_, 5% CO_2_, 37 °C).

### 4.5. Fibroblast Base Editing

The protocol was modified from Martufi et al., 2019 [57]. *FANCA* c.3934 + 2T > C patient-derived fibroblasts were washed once in PBS and centrifuged at 90× *g* for 10 min. Per condition, 2.5e5 fibroblasts were resuspended in 16 μL of Amaxa P3 Nucleofector Solution (Lonza, Basel, Switzerland, #PBP3-00675) and combined with either 2.53 μg of *B2M* Ex.1 SD or *FANCA* Ex.39 sgRNAs (Synthego, Menlo Park, CA, USA), 1.65 μg of BE4 mRNA (TriLink Biotechnologies, San Diego, CA, USA) or 10.4 μg of BE4 RNP, and 0.1 μg of eGFP mRNA (TriLink Biotechnologies, San Diego, CA, USA, #L-7201-1000), and 0.6 μL of 100 μM Alt-R Cas9 Electroporation Enhancer (IDT, Coralville, IA, USA, #1075916). The final 20 μL reaction suspensions were added to 20 μL P3 Amaxa Nucleocuvettes (Lonza, Basel, Switzerland, #PBP3-00675) and electroporated via protocol CM-138 in an Amaxa 4D-Nucleofector (Lonza, Basel, Switzerland). Fibroblasts were allowed to recover in antibiotic-free fibroblast medium under hypoxic conditions (5% O_2_, 5% CO_2_, 37 °C) for 20 min, followed by culturing in complete fibroblast medium for 5 days under hypoxic conditions (5% O_2_, 5% CO_2_, 37 °C).

### 4.6. LCL Base Editing

FA-55 LCL were washed once in PBS and centrifuged at 200× *g* for 10 min. Per condition, 1e6 LCL were resuspended in 16 μL of Amaxa SE Nucleofector Solution (Lonza, Basel, Switzerland, #PBC1-00675) and combined with either 1 μg of *B2M* Ex.1 SD or *FANCA* Ex.4 sgRNAs (Synthego, Menlo Park, CA, USA), 1.5 μg of ABE7.10max mRNA (TriLink Biotechnologies, San Diego, CA, USA) or 1.5 μg of ABE8e mRNA (TriLink Biotechnologies, San Diego, CA, USA), and 0.1 μg of eGFP mRNA (TriLink Biotechnologies, San Diego, CA, USA, #L-7201-1000), and 1.5 μL of Protector RNase Inhibitor (1:50, 40 U/μL, Roche, Basel, Switzerland, #03335399001). The final 20 μL reaction suspension was incubated at RT for 5 min and then added to 20 μL P3 Amaxa Nucleocuvettes (Lonza, Basel, Switzerland, #PBC1-00675) and electorporated via protocol EO-117 in an Amaxa 4D-Nucleofector (Lonza, Basel, Switzerland). LCLs were allowed to recover in antibiotic-free LCL medium under normoxic conditions (21% O_2_, 5% CO_2_, 37 °C) for 20 min, followed by culturing in complete LCL medium for 5 days under normoxic conditions (21% O_2_, 5% CO_2_, 37 °C).

### 4.7. Genomic Analysis

Genomic DNA was isolated from 1 × 10^6^ fibroblast or LCL 5 days post electroporation with a GeneJET Genomic Purification Kit (Thermo Scientific, Waltham, USA, #00967954). Base editing efficiency and indel formation were analyzed on the genomic level by the PCR amplification of *B2M* Ex.1 SD, *FANCA* Ex.39, or *FANCA* Ex.4 sequences. The confirmation of amplification by band size was conducted using a 1% agarose gel run at 135 V for 45 min. After purification with a QIAquick PCR Purification Kit (QIAGEN, Germantown, USA, #28104), all cultures were sent to Eurofins Corporation (Louisville, KY, USA) for the Sanger sequencing of the PCR amplicons. Subsequent analysis of the Sanger sequencing traces used the web app EditR [35] (https://baseeditr.com, accessed on 1 January 2018–1 July 2022) for base editing outcomes, and indel formation by Cas9 was assessed using Synthego ICE software (https://ice.synthego.com, accessed on 1 January 2018–1 July 2022).

### 4.8. In Vitro Base Editing Assays

All sgRNAs and primers to generate dsDNA template sequences can be found in the methods below. To form the RNP (Aldeveron, Madison, WI, USA) between BE4 and each sgRNA; 0.5 μL of BE4 protein (18.8 μg/μL) was added to 1.5 μL of respective sgRNA (1.0 μg/μL) and incubated for 20 min at 21 °C. During RNP formation, 600 ng of target dsDNA and 1.5 μL of NEB buffer 3.1 (New England BioLabs, Ipswich, USA, #B7203) were brought up to a total volume of 13 μL with nuclease-free water. After the 20 min incubation, RNP complexes were added to the dsDNA mixtures and mixed gently. Each reaction was incubated for 12 h at 37 °C. Post incubation, reactions were PCR-purified using QIAquick PCR purification kits and Sanger sequenced with the *FANCA* Ex. 39 forward and reverse primers. Sequencing was analyzed for base editing with EditR.

### 4.9. sgRNAs and PCR Primers

sgRNA or Primer Sequence

B2M Ex.1 SD

ACTCACGCTGGATAGCCTCC

FANCA Ex.39 20 nt

TGGCAAGAAACACGCTGCTG

FANCA Ex.39 21 nt

GTGGCAAGAAACACGCTGCTG

FANCA Ex.39 22 nt

AGTGGCAAGAAACACGCTGCTG

FANCA Ex.39 23 nt

GAGTGGCAAGAAACACGCTGCTG

FANCA Ex.4

CTTTGCAGGATCAAGCCTCA

B2M Ex.1 SD

fwd: TTGGAGACAGGTGACGGTCC

rev: TTATCGACGCCCTAAACTTTGTCC

FANCA Ex.39

fwd: GAGGAAATGCCCTCTTCTGT

rev: TTGACCAGTGAGCCAGTAAA

FANCA Ex.4

fwd: AAGGCATTTTAAACAGCAAG

rev: AGACGGGAGAACATACTGTG

### 4.10. Flow Cytometry

Aliquots of 2 × 10^5^ cells from B2M and zap control cultures were collected and washed twice with PBS, with centrifugation at 400× *g* for 5 min. Cells were then incubated with 5 µL TruStain FcX (BioLegend, San Diego, CA, USA, #422302) in FACS tubes at room temperature for 5 min. Without washing, cells were then incubated on ice in the dark for 20 min with 10 µL eFluor780 Fixable Viability Dye (1:500, eBioScience, San Diego, CA, USA, #65-0865-18) and 1 µL PE/Cy7 anti-human β2-microglobulin [2M2] monoclonal antibody (1:5, BioLegend, San Diego, CA, USA, #316318). After washing, cells were reconstituted in 250 µL PBS and immediately analyzed on a CytoFLEX S Flow Cytometer (Beckman Coulter, Brea, CA, USA, #C09766) using CytExpert v software. Data were analyzed using FlowJo v10 software (BD Life Sciences, Franklin Lakes, NJ, USA).

### 4.11. MMC Sensitivity Assay

*FANCA* c.3934 + 2T > C fibroblasts and *FANCA* c.3934 + 2T > C edited fibroblasts were seeded at a density of 5 × 10^5^ cells/mL in fibroblast media supplemented with MMC (0–80 nM MMC, Selleck Chemicals, Houston, TX, USA, #S8146) or DMSO (1 µL/mL, Thermo Scientific, Waltham, MA, USA, #BP231-100). Supplemented fibroblast media was doubled as appropriate and cells were counted after 12 days using a Countess™ II Automated Cell Counter (Thermo Fisher, Waltham, MA, USA, #AMQAX1000) with Countess™ Cell Counting Chamber Slides (Thermo Scientific, Waltham, MA, USA, #C10312) using Trypan Blue Stain (0.4% Thermo Scientific, Waltham, MA, USA, #T10282).

FA-55 LCL and FA-55-edited LCLs were seeded at a density of 5 × 10^5^ cells/mL in LCL media supplemented with MMC (40 µM MMC, Selleck Chemicals, Houston, USA, #S8146) or DMSO (1 µL/mL, Thermo Scientific, Waltham, MA, USA, #BP231-100). Cells were counted every other day for cell expansion and viability using a Countess™ II Automated Cell Counter (Thermo Fisher, Waltham, MA, USA, #AMQAX1000) with Countess™ Cell Counting Chamber Slides (Thermo Scientific, Waltham, MA, USA, #C10312) using Trypan Blue Stain (0.4% Thermo Scientific, Waltham, MA, USA, #T10282). Cell suspensions were mixed and LCL media supplemented with new MMC (40 µM MMC, Selleck Chemicals, Houston, TX, USA, #S8146) or DMSO (1 µL/mL, Thermo Scientific, Waltham, MA, USA, #BP231-100) was doubled every other day, moving to new plates and flasks as appropriate.

### 4.12. FANCA Western Blot

Protein extracts were isolated from 1e6 HD, FA-55, FA-55-edited, and FA-55 zap control LCLs with 50 µL Radioimmunoprecipitation Assay Buffer (150 mM NaCl, 1.0% IGEPAL^®^ CA-630, 0.5% sodium deoxycholate, 0.1% SDS, 50 mM Tris, pH 8.0) in an ice filled sonicator for 3 min. Cell lysates were centrifuged at 15,000× *g* for 15 min at 4 °C followed by supernatant collection. Quantification was conducted with a PierceTM BCA Protein Assay Kit (Thermo Scientific, Waltham, MA, USA, #23225) according to the manufacturer’s instructions with Pre-Diluted Protein Assay Standards: Bovine Serum Albumin Set (Thermo Scientific, Waltham, MA, USA, #23208) in a spectrophotometer (Biotek, #271330) read at an absorbance of 570 nm. Protein lysates (20 µg) were analyzed with an Anti-Rabbit Detection Module for Jess/Wes, Peggy Sue or Sally Sue Kit (Protein Simple, San Jose, CA, USA, #DM-001) according to the manufacturer’s instructions with an Anti-Mouse Secondary NIR Antibody (Protein Simple, San Jose, CA, USA, #043-821). After sample preparation and plate loading, final mixtures were run through 12–230 kDa Jess/Wes Separation Module, 8 × 25 capillary cartridges (Protein Simple, San Jose, CA, USA, #SM-W004) and read with a Jess Simple Western System (Protein Simple, San Jose, CA, USA, #JS-3488) between 3500–4000 V. A 1:50 dilution of Anti-FANCA/FAA rabbit polyclonal antibody (Abcam, Cambridge, UK, #ab5063) was used with a 1:50 dilution of β-Actin mouse polyclonal antibody clone 8H10D10 (Cell Signaling Technologies, Danvers, MA, USA, #3700) as a loading control.

### 4.13. FANCD2 Western Blot

HD, FA-55, FA-55-edited, and FA-55 zap control LCLs were seeded at a density of 5 × 10^5^ cells/mL in LCL media supplemented with MMC (40 nM, Selleck Chemicals, Houston, TX, USA, #S8146) or DMSO (1 µL/mL, Thermo Scientific, Waltham, MA, USA, #BP231-100) for 24 h. Protein extracts were isolated from 1 × 10^6^ cells with 50 µL Radioimmunoprecipitation Assay Buffer (150 mM NaCl, 1.0% IGEPAL^®^ CA-630, 0.5% sodium deoxycholate, 0.1% SDS, 50 mM Tris, pH 8.0) in an ice filled sonicator for 3 min. Cell lysates were centrifuged at 15,000× *g* for 15 min at 4 °C, followed by supernatant collection. Quantification was conducted with a PierceTM BCA Protein Assay Kit (Thermo Scientific, Waltham, MA, USA, #23225) according to the manufacturer’s instructions with Pre-Diluted Protein Assay Standards: Bovine Serum Albumin Set (Thermo Scientific, Waltham, MA, USA, #23208) in a spectrophotometer (Biotek, #271330) read at an absorbance of 570 nm. Cell lysates (10 µg) were loaded and run on 4–12% Bis-Tris gels (Thermo Fisher, Waltham, MA, USA, #NP0335BOX) on a PowerEase 300 W Platform (Invitrogen, Waltham, MA, USA, #PS0300) at 130 V for 4 h in the dark. Completed 4–12% Bis-Tris gels (Thermo Fisher, Waltham, MA, USA, #NP0335BOX) were transferred to PVDF membranes (Bio-Rad, #SE1M003M00) and blocked with 5% BSA/TBS-T (Sigma, St. Louis, MO, USA, #A2153) for 1 h at RT and then incubated with a 1:50 dilution of Anti-FANCD2/FAD2 rabbit polyclonal antibody (Abcam, #ab5063) in 5% BSA/TBS-T (Sigma, St. Louis, MO, USA, #A2153) overnight at 4 °C. PVDF membranes (Bio-Rad, #SE1M003M00) were then washed four times with 5% BSA/TBS-T (Sigma, St. Louis, MO, USA,#A2153) and incubated with a 1:2000 dilution of Anti-Rabbit IgG, HRP-linked Secondary Antibody (Cell Signaling Technologies, #7074) at RT for 1 h. Completed PVDF membranes (Bio-Rad, #SE1M003M00) were then washed six times with 5% BSA/TBS-T (Sigma, St. Louis, MO, USA, #A2153) and developed using a WesternBright Quantum detection kit (Advansta, San Jose, CA, USA, #K-12042). Imaging was conducted with a LICOR Odyssey (LICOR, Lincoln, NE, USA, #XF) and analyzed using Image Studio™. A 1:50 dilution of β-Actin mouse polyclonal antibody (Cell Signaling Technologies, Danvers, MA, USA #3700) was used as a loading control.

### 4.14. Statistical Analysis

The Student’s *t*-test was used to evaluate for significant differences between groups. Differences between two or more groups with one data point were evaluated by a one-way ANOVA test. Differences between three or more groups with multiple data points were evaluated by a two-way ANOVA test. Means values + SEM are shown. The levels of significance were set at *p* < 0.05. All statistical analyses were performed using GraphPad Prism 9.2.0.

### 4.15. Computational Analysis

Genes mutated in FA were determined from the literature [49]. ClinVar [58] mutations were found using the search term “((Fanconi Anemia) OR Fanconi’s Anemia OR Fanconi) NOT Invitae [Submitter]” with uncertain, likely pathogenic, and pathogenic selection. Invitae was excluded as a submitter due to a large proportion of their submissions being of somatic mutations found in cancer mutations as opposed to germline mutations in patients with FA. ClinVar mutations were filtered on mutations in the aforementioned genes determined to be mutated in FA. For BE-Hive analysis, the *FANCA* c.3934 + 2 T > C mutation and the surrounding 100 bp region was entered into the BE-Hive prediction tool using batch mode, with “Base editing outcomes among sequenced reads: DNA sequence” selected (https://www.crisprbehive.design/guide) (accessed on 2 September 2020). Please see Supplementary Files Table S1 and Data S1 for reproducible analysis in R.

## Figures and Tables

**Figure 1:**
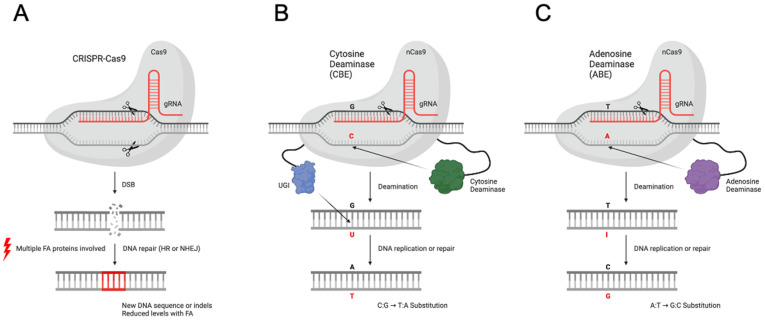
Bypassing DSB induction with base editor technology is ideal for FA gene therapy. (**A**) CRISPR-Cas9 produces DSBs that are difficult for FA-mutated cells to resolve during gene therapy; a designed sgRNA complex with Cas9 nuclease which localizes and binds to the complementary target site. Cas9 creates a DSB in the DNA that the cell resolves through the error prone NHEJ pathway or the HDR pathway, although to reduced levels in FA-mutated cells. (**B**) CBE; nCas9, which instead only nicks the opposite target stand, induces a DNA repair pathway response. The target C in the editing window is deaminated by a fused APOBEC1 protein to a U, and a fused uracil glycosylase inhibitor (UGI) protein prevents the cell from resolving this mismatch through the BER pathway. During DNA replication or repair, the U matches with an A resulting in a C/G to T/A substitution. (**C**) ABE; nCas9, which also only nicks the opposite target stand, induces a DNA repair pathway response. The target A in the editing window is deaminated by a fused TadA protein to an inosine, I. During DNA replication or repair, the I is read as a G resulting in an A/T to G/C substitution.

**Figure 2:**
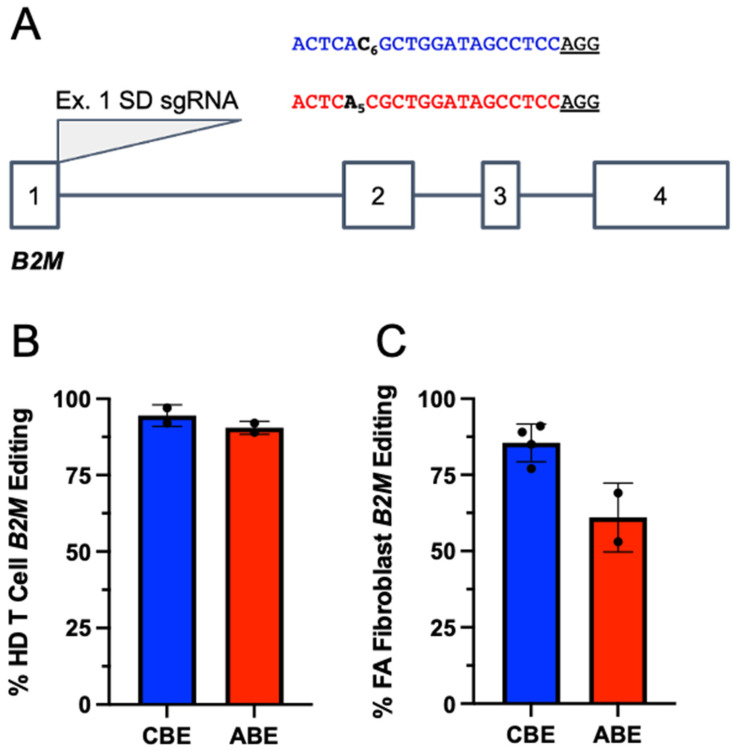
Base editors are highly functional in FA cells. (**A**) Diagram of the *B2M* genetic locus showing the four exons and three introns. Immediately following exon 1, a SD site was targeted with a sgRNA (gray triangle) in combination with CBE or ABE mRNA which causes protein KO. The sgRNA is depicted in blue showing the target C base when using CBE and in red showing the target A base when using ABE. (**B**) HD T cells were electroporated using the sgRNA shown above in combination with CBE or ABE mRNA with the Amaxa system. Data are represented as mean ± SD with *n* = 2 replicates. (**C**) FA fibroblasts were electroporated using the sgRNA shown above in combination with CBE or ABE mRNA with the Amaxa system. Data are represented as mean ± SD with *n* = 2 or *n* = 4 replicates.

**Figure 3:**
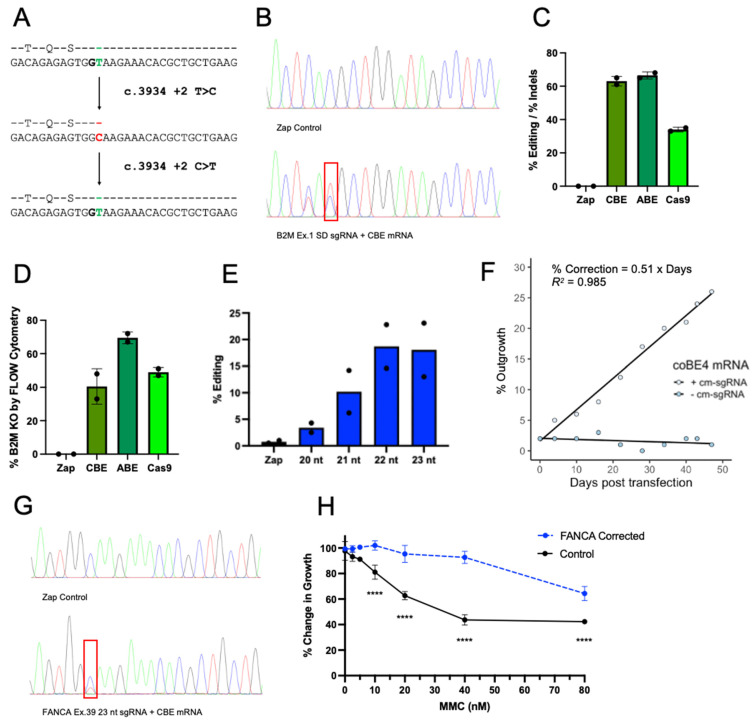
Cytosine base editing in *FANCA* c.3934 + 2T > C patient-derived fibroblasts and LCL. (**A**) Editing scheme; immediately following *FANCA* exon 39, a SD (GT) is disrupted by a + 2T > C mutation (CG). CBE directly converts C > T back to the WT sequence. (**B**) *B2M* Ex.1 SD sgRNA region chromatograms of Sanger sequencing after PCR amplification of *B2M* exon 1 using (http://baseeditr.com/, accessed on 1 January 2018–1 July 2022) [35]. (**C**) Percentage of *B2M* editing events (Kluesner and Nedveck et al. 2018) or indels (https://ice.synthego.com/#/, accessed on 1 January 2018–1 July 2022) identified by Sanger sequencing 5 days post electroporation. Data are represented as mean ± SD with *n* = 2 replicates. (**D**) Percentage of B2M protein KO as shown by flow cytometry 6 days post electroporation. (**E**) Percentage of *FANCA* editing events identified by Sanger sequencing after in vitro incubation of *FANCA* Ex. 39 amplicons, reverse FANCA Ex. 39 primers, and respective sgRNAs. Data are represented as mean ± SD with *n* = 2 replicates. (**F**) Correlation of editing (C > T) as identified by Sanger sequencing of FA fibroblasts corrected using CBE mRNA with *FANCA* c.3934 + 2T > C 22 nt sgRNA over time. Cell pellets were collected and analyzed on respective days up to 47 days in fibroblast media. (**G**) *FANCA* sgRNA region chromatograms of Sanger sequencing after PCR amplification of *FANCA* exon 39 using EditR. (**H**) MMC hypersensitivity in unedited (control) and edited FA fibroblasts (CBE mRNA + *FANCA* c.3934 + 2T > C 22 nt sgRNA) after 9 days in culture. Data are represented as mean ± SD with *n* = 3 replicates. Two-way ANOVA followed by Sidak’s multiple comparison test (**** *p* < 0.0001).

**Figure 4:**
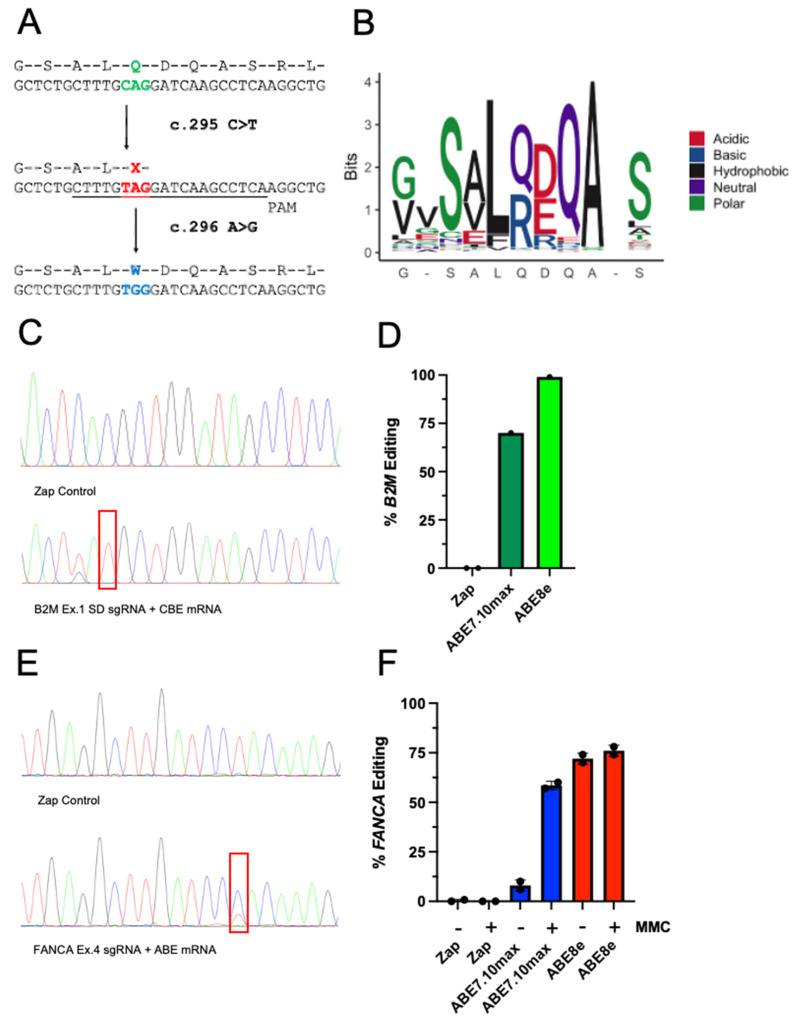
ABE editing in *FANCA* c.295C > T patient-derived LCL. (**A**) Editing scheme; In FA patient’s with the Spanish founder mutation, a pmSTOP codon (TAG) is mutated from the WT glutamine (CAG). ABE changes this pmSTOP codon (A > G) to tryptophan (TTG) resulting in translation of the *FANCA* gene. (**B**) Alignment of amino acids surrounding the *FANCA* c.295 region on exon 4 showing variability of the codon in questions across species. (**C**) *B2M* Ex.1 SD sgRNA region chromatograms of Sanger sequencing after PCR amplification of *B2M* exon 1 using EditR. (**D**) Percentage of *B2M* editing events (http://baseeditr.com/, accessed on 1 January 2018–1 July 2022) identified by Sanger sequencing 5 days post electroporation. Data are represented as mean ± SD with *n* = 2 and *n* = 1 replicates, respectively. (**E**) *FANCA* sgRNA region chromatograms of Sanger sequencing after PCR amplification of *FANCA* exon 4. Reverse complement primer sequence is shown. (**F**) Percentage of *FANCA* editing events identified by Sanger sequencing 5 days after electroporation. Data are represented as mean ± SD with *n* = 2 and *n* = 1 replicates, respectively.

**Figure 5:**
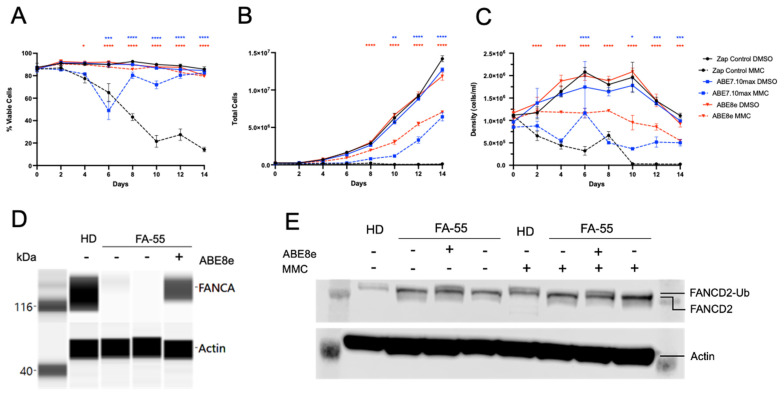
Phenotypic restoration of the FA pathway after base editing the Spanish founder mutation. MMC hypersensitivity in edited and unedited FA LCL with courts taken every other day showing cell viability (**A**), cell expansion (**B**), and cell density (**C**). All data are represented as mean ± SD with *n* = 3 replicates. (* *p* < 0.05, ** *p* < 0.001, *** *p* < 0.0005, **** *p* < 0.0001). (**D**) FANCA Western blot before and after editing showing protein product restoration using the Anti-Rabbit Detection Module for Jess/Wes. (**E**) Traditional FANCD2 Western blot run on 4–12% Bis-Tris gels and PVDF membranes. The presence of a second higher molecular weight band after base editing indicates FANCD2 monoubiquitination and thus an intact FA pathway.

**Figure 6:**
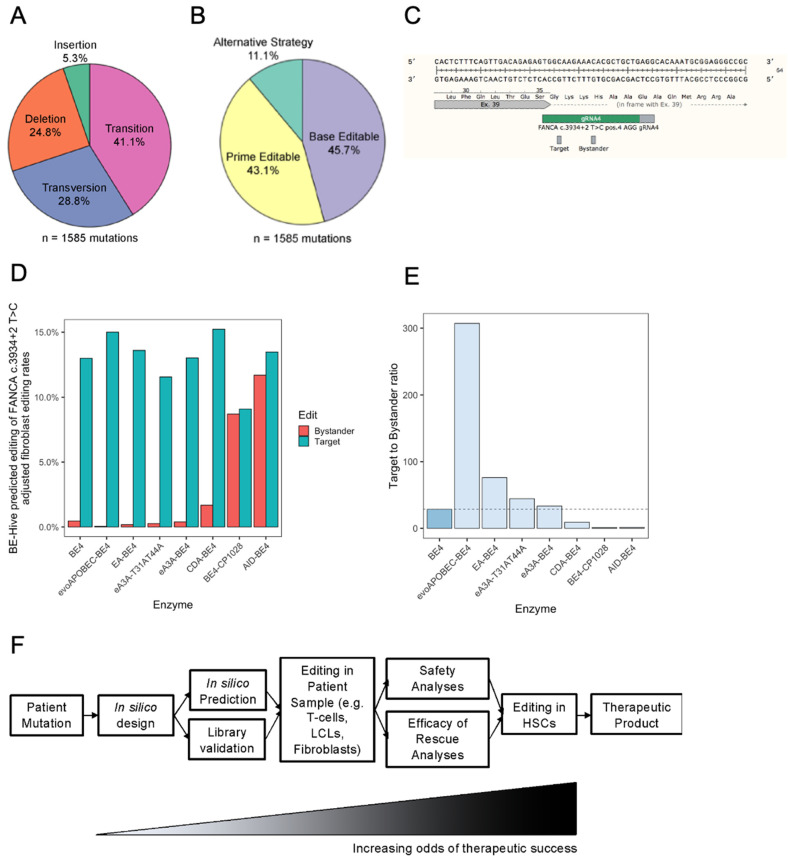
Computational assessment of the feasibility of digital editing for Fanconi Anemia mutations. (**A**) Piechart of ClinVar mutations showing the percentage of each mutation class; transition, transversion, deletion, and insertion mutations. (**B**) Piechart of ClinVar mutations correctable by digital editing strategies; base editing, prime editing. Mutations not correctable by digital editing are grouped under requiring alternative strategies. (**C**) Schematic of *FANCA* c.3934 + 2T > C locus with target and bystander bases when edited with CBE. (**D**) BE-Hive predictions of CBE efficiencies for *FANCA* c.3934 + 2T > C, adjusted for observed editing rates in fibroblast experiments (Figure 3). (**E**) Target to bystander ratio of BE-Hive-predicted editing. (**F**) Proposed pipeline for the selection and validation of patient mutation correction by digital editors.

## Data Availability

Not applicable.

## Supplementary Materials

The following supporting information can be downloaded at: https://www.mdpi.com/article/10.3390/ijms23158416/s1.
