# Supplementary material for: Correction of Fanconi Anemia Mutations Using Digital Genome Engineering
Source: Int J Mol Sci. 2022 Jul 29;23(15):8416. doi: 10.3390/ijms23158416 (PMC9369391; doi:10.3390/ijms23158416)
Supplement: Supplementary file 1 [file ijms-23-08416-s001.zip › Supp Figures S1-S3.pptx]

## Slide 1
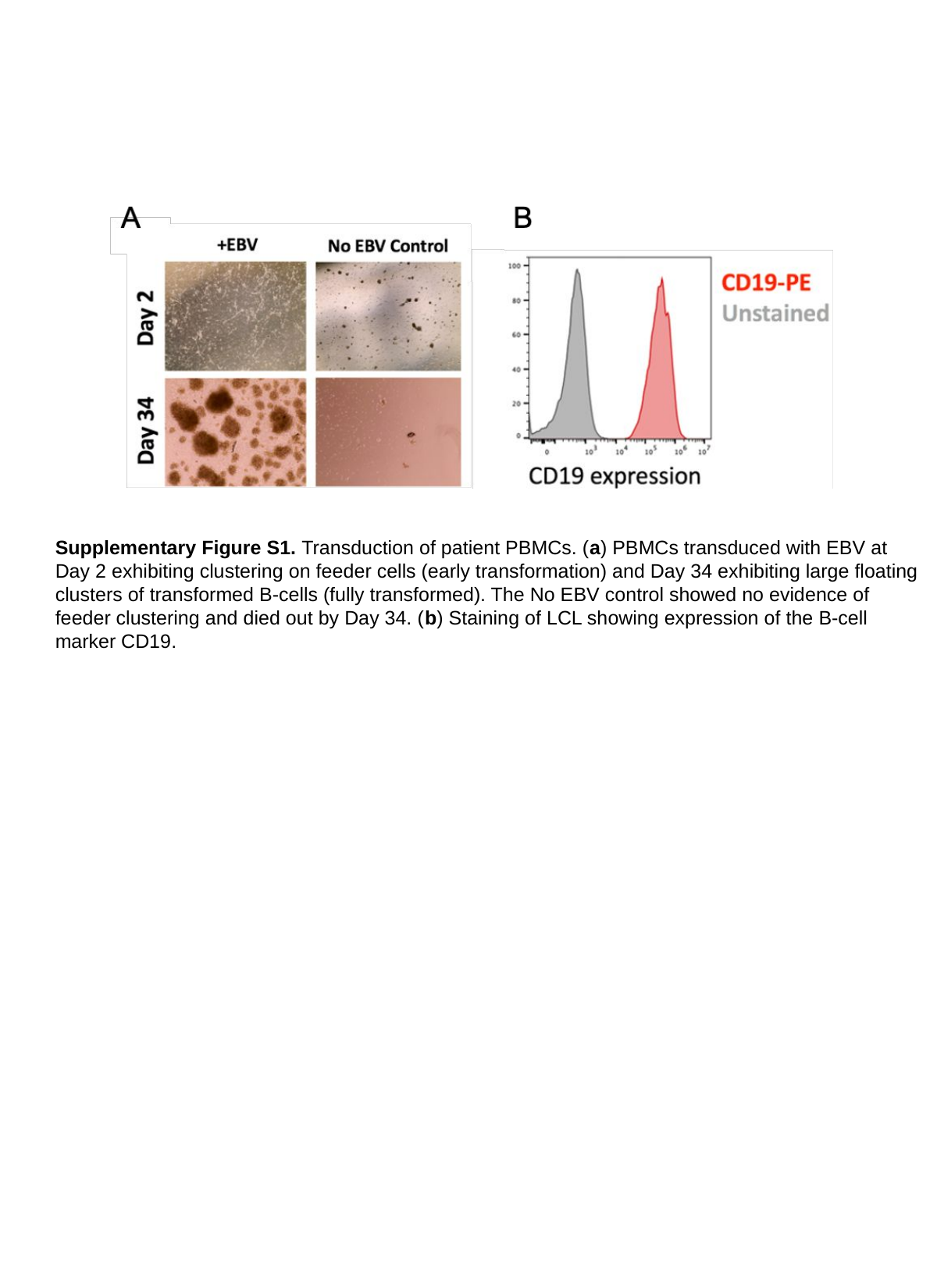

Supplementary Figure S1. Transduction of patient PBMCs. (a) PBMCs transduced with EBV at Day 2 exhibiting clustering on feeder cells (early transformation) and Day 34 exhibiting large floating clusters of transformed B-cells (fully transformed). The No EBV control showed no evidence of feeder clustering and died out by Day 34. (b) Staining of LCL showing expression of the B-cell marker CD19.

## Slide 2
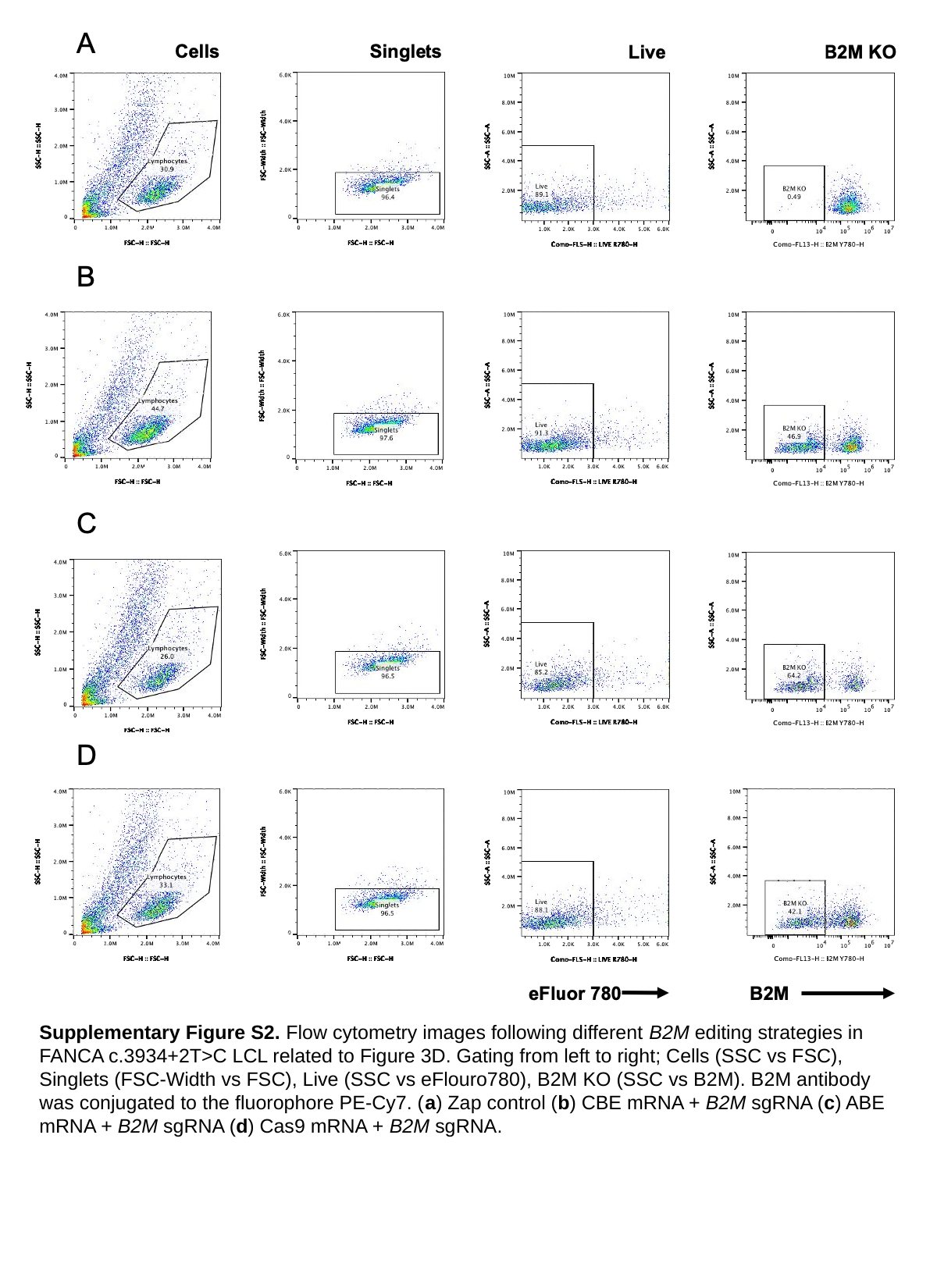

Supplementary Figure S2. Flow cytometry images following different B2M editing strategies in FANCA c.3934+2T>C LCL related to Figure 3D. Gating from left to right; Cells (SSC vs FSC), Singlets (FSC-Width vs FSC), Live (SSC vs eFlouro780), B2M KO (SSC vs B2M). B2M antibody was conjugated to the fluorophore PE-Cy7. (a) Zap control (b) CBE mRNA + B2M sgRNA (c) ABE mRNA + B2M sgRNA (d) Cas9 mRNA + B2M sgRNA.

## Slide 3
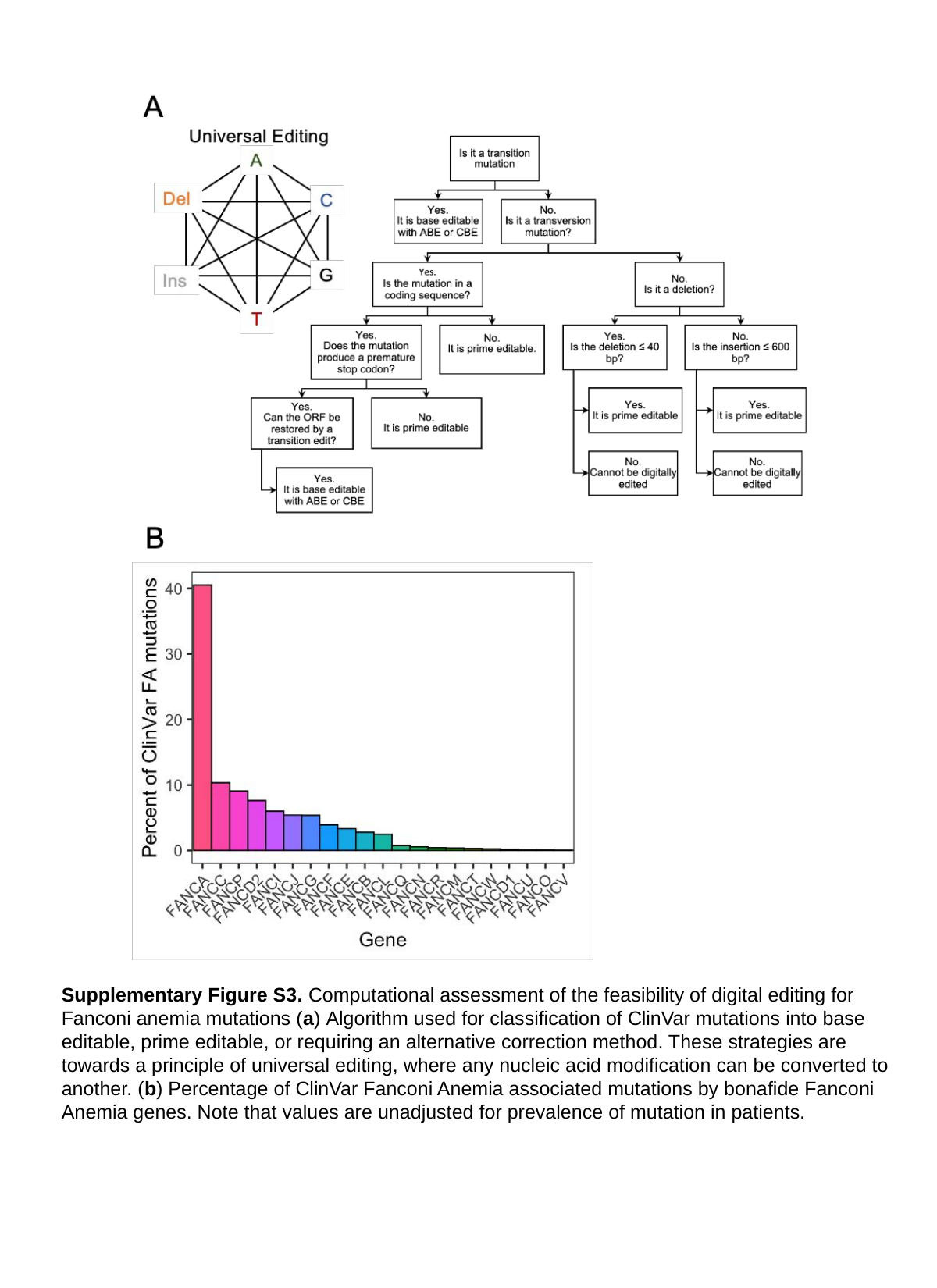

Supplementary Figure S3. Computational assessment of the feasibility of digital editing for Fanconi anemia mutations (a) Algorithm used for classification of ClinVar mutations into base editable, prime editable, or requiring an alternative correction method. These strategies are towards a principle of universal editing, where any nucleic acid modification can be converted to another. (b) Percentage of ClinVar Fanconi Anemia associated mutations by bonafide Fanconi Anemia genes. Note that values are unadjusted for prevalence of mutation in patients.

## Slide 4
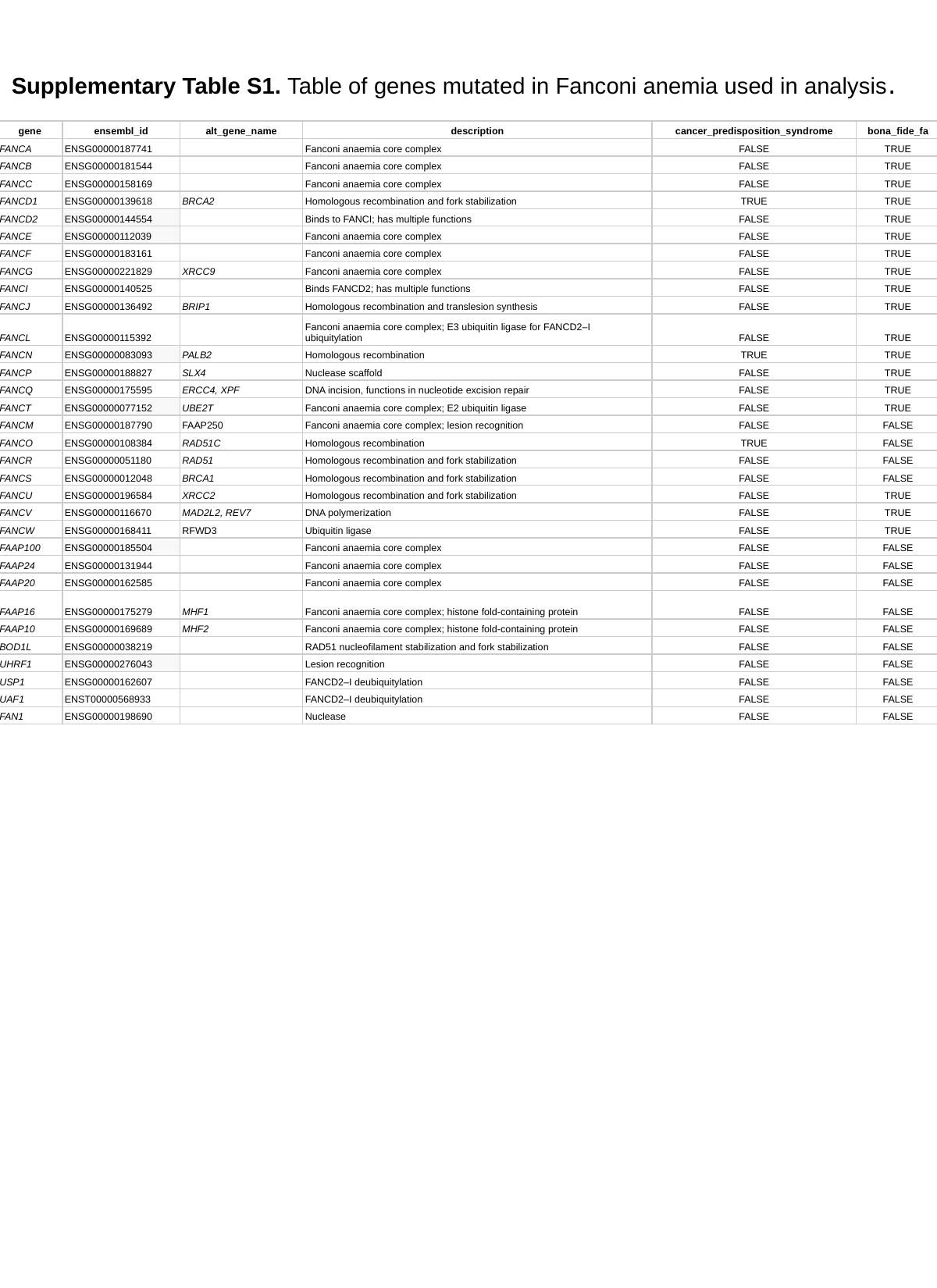

Supplementary Table S1. Table of genes mutated in Fanconi anemia used in analysis.
| gene | ensembl\_id | alt\_gene\_name | description | cancer\_predisposition\_syndrome | bona\_fide\_fa |
| --- | --- | --- | --- | --- | --- |
| FANCA | ENSG00000187741 | | Fanconi anaemia core complex | FALSE | TRUE |
| FANCB | ENSG00000181544 | | Fanconi anaemia core complex | FALSE | TRUE |
| FANCC | ENSG00000158169 | | Fanconi anaemia core complex | FALSE | TRUE |
| FANCD1 | ENSG00000139618 | BRCA2 | Homologous recombination and fork stabilization | TRUE | TRUE |
| FANCD2 | ENSG00000144554 | | Binds to FANCI; has multiple functions | FALSE | TRUE |
| FANCE | ENSG00000112039 | | Fanconi anaemia core complex | FALSE | TRUE |
| FANCF | ENSG00000183161 | | Fanconi anaemia core complex | FALSE | TRUE |
| FANCG | ENSG00000221829 | XRCC9 | Fanconi anaemia core complex | FALSE | TRUE |
| FANCI | ENSG00000140525 | | Binds FANCD2; has multiple functions | FALSE | TRUE |
| FANCJ | ENSG00000136492 | BRIP1 | Homologous recombination and translesion synthesis | FALSE | TRUE |
| FANCL | ENSG00000115392 | | Fanconi anaemia core complex; E3 ubiquitin ligase for FANCD2–I ubiquitylation | FALSE | TRUE |
| FANCN | ENSG00000083093 | PALB2 | Homologous recombination | TRUE | TRUE |
| FANCP | ENSG00000188827 | SLX4 | Nuclease scaffold | FALSE | TRUE |
| FANCQ | ENSG00000175595 | ERCC4, XPF | DNA incision, functions in nucleotide excision repair | FALSE | TRUE |
| FANCT | ENSG00000077152 | UBE2T | Fanconi anaemia core complex; E2 ubiquitin ligase | FALSE | TRUE |
| FANCM | ENSG00000187790 | FAAP250 | Fanconi anaemia core complex; lesion recognition | FALSE | FALSE |
| FANCO | ENSG00000108384 | RAD51C | Homologous recombination | TRUE | FALSE |
| FANCR | ENSG00000051180 | RAD51 | Homologous recombination and fork stabilization | FALSE | FALSE |
| FANCS | ENSG00000012048 | BRCA1 | Homologous recombination and fork stabilization | FALSE | FALSE |
| FANCU | ENSG00000196584 | XRCC2 | Homologous recombination and fork stabilization | FALSE | TRUE |
| FANCV | ENSG00000116670 | MAD2L2, REV7 | DNA polymerization | FALSE | TRUE |
| FANCW | ENSG00000168411 | RFWD3 | Ubiquitin ligase | FALSE | TRUE |
| FAAP100 | ENSG00000185504 | | Fanconi anaemia core complex | FALSE | FALSE |
| FAAP24 | ENSG00000131944 | | Fanconi anaemia core complex | FALSE | FALSE |
| FAAP20 | ENSG00000162585 | | Fanconi anaemia core complex | FALSE | FALSE |
| FAAP16 | ENSG00000175279 | MHF1 | Fanconi anaemia core complex; histone fold-containing protein | FALSE | FALSE |
| FAAP10 | ENSG00000169689 | MHF2 | Fanconi anaemia core complex; histone fold-containing protein | FALSE | FALSE |
| BOD1L | ENSG00000038219 | | RAD51 nucleofilament stabilization and fork stabilization | FALSE | FALSE |
| UHRF1 | ENSG00000276043 | | Lesion recognition | FALSE | FALSE |
| USP1 | ENSG00000162607 | | FANCD2–I deubiquitylation | FALSE | FALSE |
| UAF1 | ENST00000568933 | | FANCD2–I deubiquitylation | FALSE | FALSE |
| FAN1 | ENSG00000198690 | | Nuclease | FALSE | FALSE |

## Slide 5
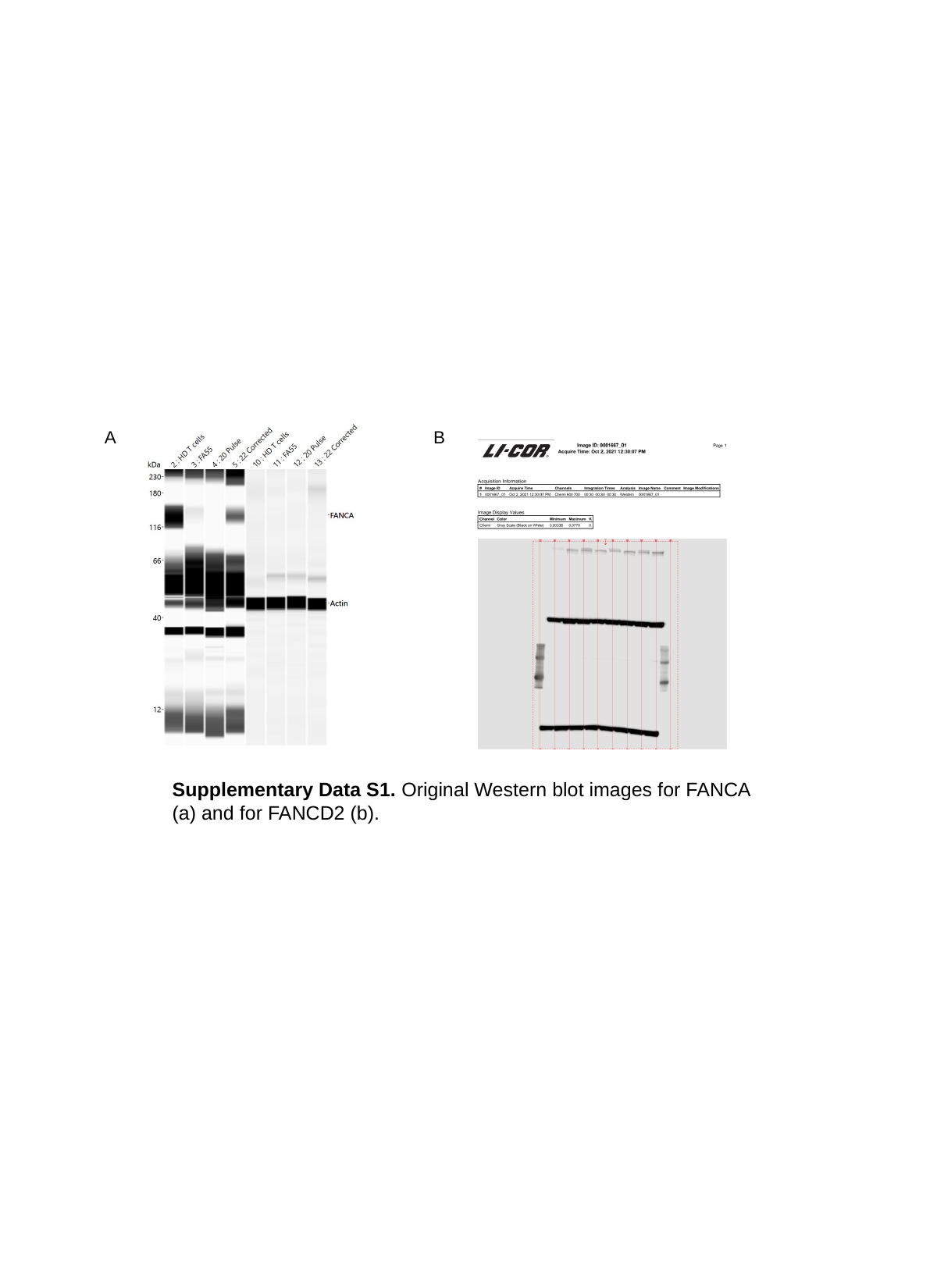

A
B
Supplementary Data S1. Original Western blot images for FANCA (a) and for FANCD2 (b).
